# Professional Perspicuity

**Published:** 1869-08-15

**Authors:** 


### EDITORIAL NOTES.

### *Professional Perspicuity.*

In an original communication in the July number of the *Medical Examiner*, upon the subject of tetanus occurs the following passage:

"L. B., a young man aged 22, was out hunting near Calumet; while in the act of loading his gun, or after it was loaded and capped, his dog, a fine, noble looking beast, sprang upon him; in passing his paws to the ground they struck the cock, and the contents were immediately lodged in the fore-arm and the other hand."

Such, it appears, was the source of a case of tetanus, which, the writer kindly informs us, in another place, "is an affection of the excito-motor nerves."

The very ambiguous phraseology of the above quotation suggests three queries, viz:

1st. What was the nature of those "*contents?*"

2d. Of what were they the "*contents?*"

3d. What would be the effect of "lodging them immediately in the forearm and the other hand?"

The most immediate effect appears to have been the decision "to give him the benefit (?) of *council;*" (he was not even allowed the "benefit of a doubt"), and the next was "amputation," of what the writer does not condescend to inform us further than to refer to it as "the operation, which is of no interest here to describe." The next consequence in order appears to have been "tetanus," during which "the opisthotonos which was *permanent* was *at times increased* by violent *clonic spasms* of the same muscles." Paradoxical!

To the administration of "three drops of croton oil" and a "fly blister laid on the whole course of the spinal column" succeeded that of "two grains of sulphate of morphia every hour." The consequence of which seems to have been that "in the evening of the same day I found the symptoms very much increased in severity." For which result surely neither the patient nor the medicine should be blamed.

Subsequently to this the patient was subjected to the influence of the Calabar bean, which, after repeated administrations, both by the mouth and sub-cutaneously, was continued in the latter form at intervals of "two hours" in doses "of half a grain of sulphate of morphia," and this "after the tetanic spasms had subsided."

Upon the development of "poisonous symptoms" the use of the bean was discontinued, and upon their subsidence "I ordered two grains of morphia by the mouth every three hours." "From that time forward he seemed to get better, but I still continued the use of the bean in one third grain doses, for two weeks."

The above is a brief review of one of the most remarkable cases in its origin, treaiment and results which we have ever had the good fortune to encounter. It is greatly to be regretted that the author did not permit an inspection of the case by some physicians of the city, or at least given the name and residence of his fearfully afflicted patient in order that the *facts* of a case so remarkable might be generally understood in the professional community.

In the interests of science we hope that the three queries prefixed will be definitely answered.

